# Creation of a Didactic Clinical Pharmacology Lecture Series for Internal Medicine Residents

**DOI:** 10.7759/cureus.42090

**Published:** 2023-07-18

**Authors:** Alice N Hemenway, Laura Meyer-Junco, Mohammed I Ahmed Khan, Mario Affinati

**Affiliations:** 1 Department of Pharmacy Practice, University of Illinois at Chicago, Rockford, USA; 2 Department of Medicine, Mercyhealth, Rockford, USA; 3 Department of Medicine, University of Illinois at Chicago, Rockford, USA

**Keywords:** lectures, residents, pharmacology, didactic, internal medicine residency

## Abstract

Introduction: One of the noted areas of weakness for internal medicine residents is pharmacology. However, there is little data documenting the creation and effect of a comprehensive pharmacology didactic program. Our goal was to create a two-year clinical pharmacology didactic program focused on areas of stated weakness and to evaluate this program for an increase in knowledge and prescribing confidence of the participants.

Methods: From August 2020 to June 2022, a two-year pharmacology program was developed, which included 20 didactic lectures on a variety of topics. Pre- and post-tests were given for 15 of the lectures, and four surveys were given, two during each year of the program. Four questions on each survey were the same and asked about confidence in choosing an appropriate medication based on current guidelines, patient-specific factors, primary literature, and pharmacokinetics.

Results: Over the two years, participation in the pharmacology sessions ranged from 17 to 29 residents (65-74% of the residency class). The average pre- and post-test scores increased by an average of 25.1%, which was a statistically significant increase (p<0.001, 95% CI [17.5, 32.8]). A Kruskal-Wallis H test showed a statistically significant difference in resident-reported confidence adjusting medications based on primary literature between the different survey groups, χ2 = 9.871, p = 0.02.

Conclusion: A two-year, didactic pharmacology program improved the knowledge of resident participants and confidence in their ability to choose an appropriate medication based on primary literature.

## Introduction

One of the areas where internal medicine residents and house staff often struggle is pharmacology [1,2]. Moulson et al. found that residents in all years of post-graduate training selected warfarin over direct-acting oral anticoagulants in a clinical scenario, which is discordant with national guidelines [2]. Internal Medicine residents often acknowledge pharmacology topics as weaknesses, with confidence issues shown in studies evaluating dose adjustments in chronic kidney disease and pharmacogenomics [3,4].

Pharmacology is a part of the medical school curriculum but is not a traditional part of residency training. Given our awareness of this weakness, it is surprising that few programs incorporate a pharmacology didactic program into training. Pharmacists are well-established clinical partners and are often involved in internal medicine rounding teams. Jorgenson et al. found in a qualitative review that pharmacists were valued as part of family medicine residency programs [5]. However, few programs had a formal pharmacology curriculum or formal evaluation of the pharmacist's role on their team [5]. In a Canadian national survey, 83.9% of pharmacist respondents reported that they provided some educational sessions but not in a formal pharmacotherapy curriculum [6]. Vairy et al. described a two-hour lecture provided by a pharmacist to pediatric residents. However, it was deemed insufficient to reduce errors in prescribing [7].

Our goal was to create a two-year clinical pharmacology didactic program focused on areas of noted weakness and to evaluate this program using pre- and post-tests as well as a longitudinal survey for an increase in knowledge and prescribing confidence by the residents.

## Materials and methods

Setting and participants

The program was implemented in a 194-bed secondary, teaching, community hospital. The hospital has an internal medicine residency with 13 residents in each class. The subjects were residents in all years of study who attended weekly half-day didactic sessions. The two-year clinical pharmacology didactic program was implemented in August 2020 and ran through June 2022, and then another two-year program was restarted in August 2022.

Interventions

The internal medicine residency program was started at the hospital in 2019. When the program was created, a half-day of didactic lectures was built into the residents’ schedules. Initially, these were mostly hour-long talks from physician specialists with the goal of preparing students for board certification. In 2020, in response to a concern about the residents’ confidence regarding pharmacology topics, a dialogue was started between the residency directors and two university-affiliated pharmacists to create a formal, didactic, pharmacology program.

The development of this program was based on the hypothesis that a structured didactic program on clinical pharmacology provided by two pharmacists with teaching experience would increase the knowledge and confidence of the internal medicine residents in clinical pharmacology topics. The program planning was done using a constructivist approach and focused on selecting diseases and medications that the residents most often encountered and providing this information using a case-based method.

A mission statement, as well as goals and objectives, was developed as a group based on published data and the WHO “Key Clinical Pharmacology Services in Patient Care,” as seen in Table 1 [8]. Individual lecture topics were developed by the pharmacists based on a survey of requested topics from current residents and the WHO “Clinical Pharmacology Core Knowledge and Understanding” [8]. The objectives were then mapped to each lecture, as seen in Table 2. Topics such as medication errors and drug interactions were included within the disease-state-based topics. Guest speakers were utilized for topics that the pharmacists thought would be better provided by content specialists. These included critical care sedation and blood pressure management; fluids, electrolytes, and nutrition; pharmacogenomics; and psychiatric medications.

**Table 1:** Goals and objectives ADME: absorption, distribution, metabolism, and excretion, IM: internal medicine

Goal	Objective A	Objective B	Objective C	Objective D
1. Critically evaluate new and old medications for various disease states, with a focus on risk/benefit analyses.	1A. Specify the pharmacologic mechanisms for medications used in common IM diseases.	1B. Evaluate the comparative efficacy of different medications used to treat common IM diseases.	1C. Incorporate pharmacoeconomic evaluation data, drug use evaluations, and prescribing management within a health system into prescribing decisions.	
2. Apply pharmacokinetic and pharmacodynamics principles when selecting an appropriate medication dosage.	2A. State how various ADME considerations affect the choice of drug and dose selection.	2B. Appropriately select the best dose regimen for an antimicrobial based on pharmacodynamic principles.	2C. Describe how pharmacokinetic and pharmacodynamic drug interactions affect drug selection and dose.	
3. Independently search and evaluate drug information from primary and tertiary resources.	3A. Demonstrate primary literature evaluation skills for various study methodologies.	3B. Compare and contrast tertiary medication information resources.	3C. Assess and incorporate guidelines into clinical practice.	
4. Select the best medication, applying information regarding inter-individual response differences.	4A. State how differences in pediatrics and geriatrics differences in pharmacokinetics and pharmacodynamics affect drug selection and dose.	4B. Describe how medication adherence, affordability, and access issues can affect inter-individual response to medications.	4C. Assess how pharmacogenomics can be integrated into patient practice and optimize prescribing.	
5. Assess a medication regimen for effectiveness and adverse effects.	5A. Describe efficacy monitoring parameters for medications used in common IM diseases.	5B. State adverse effect monitoring for medications used in common IM diseases.	5C. Demonstrate proper dose adjustment for medications utilizing therapeutic drug monitoring.	5D. Show awareness of differential risk for adverse effects in patients with altered pharmacokinetic properties or multi-drug regimens.

**Table 2:** Lecture topics and mapped objectives ADME: absorption, distribution, metabolism, elimination, ID: infectious diseases, TDM: therapeutic drug monitoring, CKD: chronic kidney disease, AKI: acute kidney injury

Topic	Mapped objective
ADME: absorption/distribution	2A, 2B, 2C, 3B, 5C
ADME: metabolism/elimination	2A, 2B, 2C
ID: antimicrobial coverage/resistance mechanisms/pharmacodynamics	1A, 1B, 2B, 2C, 5A, 5B, 5D
Pain management	1B, 2A, 4A, 5A, 5B, 5D
Literature evaluation techniques and resources	3A, 3B
Medication access/adherence/affordability	1C, 4B
Critical care medications	1A, 1B, 2C, 3C, 5A, 5B, 5D
Drug therapy in geriatric patients	1B, 2A, 2C, 4A, 5D
TDM	2A, 2B, 2C, 5C
Nausea pharmacotherapy	1A, 1B, 1C, 3C, 5A, 5B
Fluids, electrolytes, and nutrition	1A, 1B, 2C, 3C, 5A, 5B, 5D
ADME: refresher	2A, 2B, 2C, 3B, 5C
Pharmacogenomics	1C, 4C
ID/pharmacodynamics/TDM refresher	1A, 1B, 2A, 2B, 2C, 5C
Medication allergy and adverse effect assessment	3B, 5B, 5D
Psychiatric medications	1B, 2A, 3C, 5A, 5B
Antifungals, antivirals	1A, 1B, 1C, 2A, 3C, 4A, 5B
Diabetes mellitus	1A, 1B, 4A, 4B, 5A, 5B
Heart failure	1A, 1B, 4A, 5A, 5B, 5D
CKD: dose adjustments in renal replacement; medication-related AKI	2A, 2C, 5B, 5C, 5D

At the start of the program, each presentation was 120 minutes in length and was given once a month. However, over time this was shortened, first to 90 minutes and then to 60 minutes based on feedback from residents. Instead of longer sessions, if needed, topics were broken into two 60-minute sessions provided within the same month. Each lecture was PowerPoint-based, and the use of interactive questions and patient cases were encouraged. After a mid-program review, more emphasis was given to increasing both the interactivity and the patient cases in each lecture.

Outcomes measured

An increase in knowledge was assessed by a pre- and post-test for 15 of the lectures. Each lecture provided by the two pharmacists included a brief three to five-question pre-test and a post-test. The lectures provided by guest lecturers did not include pre- and post-tests. The same questions were asked in the pre- and post-tests to eliminate the risk of differences in question difficulty. The questions were developed by the two pharmacists who have additional training in question writing as well as experience in writing questions for pharmacist education, post-graduate pharmacy continuing education, and pharmacist board certification.

The acceptance and effect of the program were assessed by four surveys, given twice yearly for the two years of the program. The Likert-style survey questions were created by one of the pharmacists who have additional training in survey development and followed the process suggested for graduate medical education by Magee et al. [9]. A draft of the survey was reviewed for clarity and readability by a group of five hospital pharmacists. Each survey was administered as an anonymous paper survey at the end of the January and June lectures each year for both years of the program.

The surveys consisted of five sections, the first two sections included questions that changed each survey and were focused on the confidence and knowledge gained from each lecture. The third section of each survey was consistent between each survey and included four questions assessing the residents' confidence in choosing an appropriate medication based on current guidelines, patient-specific factors, primary literature, and pharmacokinetics. The third section also included one question assessing how often the residents utilized information from the clinical pharmacology didactic lectures. The fourth section of the survey included two open-ended questions asking about additional topics they would like the program to cover and any suggestions for the program. The final section was baseline characteristics and consisted of just a single question on post-graduate year to maintain resident anonymity.

In addition to the surveys, after the first year of the program, a residency panel was convened to discuss possible improvements to the didactic lectures, including the clinical pharmacology program. Changes made to the program based on this discussion include the shortening of lectures and utilizing multiple lectures in a month if needed to cover the material. There also was a renewed focus on providing case-based lectures and using interactive teaching methods. Finally, there was a list of board certification preparatory materials provided to the pharmacists so that these could be used to better tailor the content to what the residents will be tested on for board certification.

Analysis of the outcomes

Changes in the percentage of correct responses on the pre- and post-tests were evaluated using descriptive statistics and paired t-tests. Changes in the Likert-scale survey results were evaluated using descriptive statistics, the Kruskal-Wallis H test, and post-hoc Mann-Whitney U test for intergroup comparison. SPSS Statistics version 28.0 (IBM Corp. Released 2021. IBM SPSS Statistics for Windows, Version 28.0. Armonk, NY: IBM Corp) was utilized for statistical analysis.

IRB statement

This was determined to be exempt from review by the facility’s institutional review board (349).

## Results

Over the two years, participation in the pharmacology sessions ranged from 17 to 29 residents (65-74%). During the first year, there were two classes of residents (26 total), and during the second year, there were three classes of residents (39 total). Pre- and post-tests were given to each of the participants for 15 of the 20 sessions. Average scores for the pre-tests ranged from 32.3% to 71.0%, with a mean of 53.0% (SD= 10.6%), as seen in Table 3. Average scores for the post-tests ranged from 65.3% to 96.0%, with a mean of 78.1% (SD= 10.2%). The average scores increased by an average of 25.1% in the post-test group, which was a statistically significant increase (p<0.001, 95% CI [17.5, 32.8]).

**Table 3:** Knowledge changes in pre- and post-tests ADME: absorption, distribution, metabolism, elimination, ID: infectious diseases, TDM: therapeutic drug monitoring, CKD: chronic kidney disease, AKI: acute kidney injury

Topic	Pre-test average correct (%)	Post-test average correct (%)	Increase in average correct (%)
ADME: absorption/distribution	55.7	73.7	18
ADME: metabolism/elimination	37.2	65.3	28.1
ID: antimicrobial coverage/resistance mechanisms/pharmacodynamics	52.6	94.2	41.6
Pain management	52.9	87.7	34.8
Literature evaluation techniques and resources	59.3	71.7	12.4
Medication access/adherence/affordability	66.7	75	8.3
Drug therapy in geriatric patients	57.1	67	9.9
TDM	41.7	71	29.3
Nausea pharmacotherapy	32.3	89	56.7
ADME: refresher	46.8	73	26.2
ID/pharmacodynamics/TDM refresher	52.7	89	36.3
Medication allergy and adverse effect assessment	47.9	75.4	27.5
Antifungals, antivirals	62	69	7
Diabetes mellitus	71	96	25
CKD: dose adjustments in renal replacement; medication-related AKI	59	75	16
Total	53	78.1	25.1 (p<0.001, 95% CI [17.5, 32.8])

The total number of surveys completed was 43-15 for survey one, 12 for survey two, seven for survey three, and nine for survey four. The overall response rate was 50% (43 of 86 total lecture attendees) and ranged from 29% to 88% for each survey. Each of the four surveys asked questions about the knowledge and confidence gained from each lecture. These questions were unique to each survey. The percentage of participants who answered “positive impact” or “extreme positive impact” for the lecture-specific confidence and knowledge questions was an average of 64.8%. Each survey also asked how often the residents utilized the information from the lectures. The lecture information was used at least once a month by an average of 79.1% of survey respondents.

Four questions were asked on every survey regarding confidence in choosing an appropriate medication based on current guidelines, patient-specific factors, primary literature, and pharmacokinetics. An average of 64.2% of respondents answered “fairly confident” or “very confident” to all of these questions. The lowest confidence was in adjusting based on pharmacokinetics (55.8%), and the highest was in adjusting based on patient-specific factors (70.7%). These results fluctuated over time, with the highest confidence rates reported in the final survey, as seen in Table 4.

**Table 4:** Survey results: the percentage that answered “fairly confident” or “very confident”

Survey	Question 1: confidence adjusting based on guidelines	Question 2: confidence adjusting based on patient-specific factors	Question 3: confidence adjusting based on primary literature	Question 4: confidence adjusting based on pharmacokinetics
1. Winter 2020	73.3	73.3	66.7	66.7
2. Spring 2021	50	50	33.3	41.7
3. Winter 2021	57	71	71	29
4. Spring 2022	89	89	100	67
Total	68.3	70.7	65.1	55.8

The only statistically significant increase in the survey data was for confidence adjusting based on primary literature. A Kruskal-Wallis H test showed a statistically significant difference in confidence adjusted based on primary literature between the different survey groups, χ2 = 9.871, p = 0.02, with a mean rank confidence score of 23.17 for survey one, 28.79 for survey two, 18.29 for survey three, and 13.89 for survey four. A score of one was assigned to “very confident” and ranged down to a five assigned to “not confident,” with a lower mean rank indicating higher confidence. A posthoc Mann-Whitney U test found that the increase in confidence between surveys one and four was statistically significant (p= 0.015), as was the increase between surveys two and four (p= 0.006).

## Discussion

Our data indicates that the participants’ knowledge increased from each of the lectures, and their confidence in choosing an appropriate medication based on primary literature increased. The data did not show an increase in confidence in choosing an appropriate medication due to current guidelines, patient-specific factors, or pharmacokinetics.

To help control the quality of the lectures, the majority of lectures were created and presented by two university-affiliated pharmacists. Each lecture took approximately eight hours to create, and each pharmacist created approximately eight lectures over the two years. For large health systems with multiple clinical pharmacy specialists, one option to reduce this workload could be to share this responsibility with a larger number of pharmacists. However, an increase in lecturers would likely require a process to assure consistency and quality. In addition, each of the outside lecturers was paid a small stipend ($200) for the creation and presentation of their lectures. This should be included in any proposed didactic series in order to provide expertise that resides outside the facility.

One possible limitation of the study was the number of survey respondents. It is also unclear why the spring 2021 respondent rate was lower than the other surveys. The average response rate of 50% is good and is similar to the rate seen in other curricular assessments but may have still affected the overall results [10,11]. One possible reason for the difference in response rate compared to overall attendance could be the long survey format since each survey was five pages. Although completion of the survey was done at the end of the lecture in order to improve the response rate, it is possible that fatigue from the didactic session played a part in the reduced levels of completion.

Another possible limitation was the use of the same questions within the pre- and post-tests. Although this was done to decrease any bias due to pre- and post-test question difficulty differences, it is possible that question familiarity could have affected the results. In addition, the pre- and post-tests and surveys focused on assessing a change in knowledge and self-reported confidence, and direct application to patient care was not evaluated. Future research areas include evaluating changes in medication use or clinical outcomes related to lectures or board exam results.

Our program appears to be the first comprehensive didactic clinical pharmacology series described in the literature. A PubMed search using the combination of National Library of Medicine medical subject headings (MeSH) "internal medicine," "internship and residency," and “pharmacology, clinical" found only four results [12-15]. Of these results, two did not describe a clinical pharmacology curriculum [13,15]. One described a clinical pharmacology consult service, and the other described an oncology pharmacy residency program [13,15]. The other two results focused on specific areas of pharmacology: an interdisciplinary pain curriculum and a comprehensive cardiology program [12,14].

Reflecting on the implementation of the program, we underestimated the value of resident feedback to refine the topic selection and presentation method. Each of the surveys provided new topics and suggestions to optimize learning. Topics suggested based on this feedback that were not included in the original schedule included antifungal and antiviral medications, psychiatric medications, critical care support, and medications for nausea and vomiting. There was also a change in the length of the lecture, as well as a continued focus on case-based and interactive lectures. Incorporating residents into the design and structure of a pharmacology didactic program is necessary for optimal success.

## Conclusions

A two-year, didactic pharmacology program improved the knowledge and confidence of resident participants. Specifically, an increase in knowledge was demonstrated for each of the post-tests, with an average increase of 25.1%, which was statistically significant. This also translated into a self-reported increase in confidence in choosing a medication based on primary literature. Although the didactic sessions required a considerable amount of preparation from the pharmacist educators, this could be lessened by using a broader group of pharmacists.
